# Screening for differentially expressed microRNAs in BALF and blood samples of infected COVID‐19 ARDS patients by small RNA deep sequencing

**DOI:** 10.1002/jcla.24672

**Published:** 2022-09-27

**Authors:** Reza Najafipour, Davood Mohammadi, Zohreh Estaki, Kiana Zarabadi, Manijeh Jalilvand, Sahar Moghbelinejad

**Affiliations:** ^1^ Genetics Research Center The University of Social Welfare and Rehabilitation Science Tehran Iran; ^2^ Department of Surgery, School of Medicine Qazvin University of Medical Sciences Qazvin Iran; ^3^ Department of Pediatric Dentistry, School of Dentistry Qazvin University of Medical Sciences Qazvin Iran; ^4^ Department of Medical Genetics, School of Medicine Tarbiat Modares University Tehran Iran; ^5^ Research Institute for Prevention of Non‐Communicable Diseases, Cellular and Molecular Research Centre Qazvin University of Medical Sciences Qazvin Iran

**Keywords:** COVID‐19, gene ontology, microRNAs, respiratory distress syndrome, RNA sequencing

## Abstract

**Background:**

The pandemic COVID‐19 has caused a high mortality rate and poses a significant threat to the population of the entire world. Due to the novelty of this disease, the pathogenic mechanism of the disease and the host cell's response are not yet fully known, so lack of evidence prevents a definitive conclusion about treatment strategies. The current study employed a small RNA deep‐sequencing approach for screening differentially expressed microRNA (miRNA) in blood and bronchoalveolar fluid (BALF) samples of acute respiratory distress syndrome (ARDS) patients.

**Methods:**

In this study, BALF and blood samples were taken from patients with ARDS (*n* = 5). Control samples were those with suspected lung cancer candidates for lung biopsy (*n* = 3). Illumina high‐throughput (HiSeq 2000) sequencing was performed to identify known and novel miRNAs differentially expressed in the blood and BALFs of ARDS patients compared with controls.

**Results:**

Results showed 2234 and 8324 miRNAs were differentially expressed in blood and BALF samples, respectively. In BALF samples, miR‐282, miR‐15‐5p, miR‐4485‐3p, miR‐483‐3p, miR‐6891‐5p, miR‐200c, miR‐4463, miR‐483‐5p, and miR‐98‐5p were upregulated and miR‐15a‐5p, miR‐548c‐5p, miR‐548d‐3p, miR‐365a‐3p, miR‐3939, miR‐514‐b‐5p, miR‐513a‐3p, miR‐513a‐5p, miR‐664a‐3p, and miR‐766‐3p were downregulated. On the contrary, in blood samples miR‐15b‐5p, miR‐18a‐3p, miR‐486‐3p, miR‐486‐5p, miR‐146a‐5p, miR‐16‐2‐3p, miR‐6501‐5p, miR‐365‐3p, miR‐618, and miR‐623 were top upregulated miRNAs and miR‐21‐5p, miR‐142a‐3p, miR‐181‐a, miR‐31‐5p, miR‐99‐5p, miR‐342‐5p, miR‐183‐5p, miR‐627‐5p, and miR‐144‐3p were downregulated miRNAs. Network functional analysis for Gene Ontology (GO) and Kyoto Encyclopedia of Genes and Genomes (KEGG), in ARDS patients' blood and BALF samples, showed that the target genes were more involved in activating inflammatory and apoptosis process.

**Conclusion:**

Based on our results, the transcriptome profile of ARDS patients would be a valuable source for understanding molecular mechanisms of host response and developing clinical guidance on anti‐inflammatory medication.

## Graphical abstract

In this research, BALF and blood samples of patients with ARDS were investigated. Data from miRNA experiments were obtained from 3 normal and 5 infected samples. Total numbers of differentially expressed miRNAs in BALF was 8223 and in blood was 2223, which in BALF 825 were upregulated and 432 were downregulated. In blood samples, 543 miRNAs were upregulated and 223 miRNA downregulated. miRTarBase results for 40 high‐score differentially expressed miRNAs showed 232 target mRNA, which have 5872 interactions. The knowledge base was further validated with wet‐lab experiment, where we analyzed the correlation between some miRNAs and their target genes.

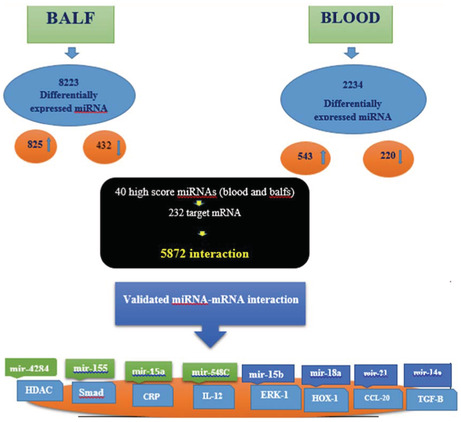

## 1 INTRODUCTION

Since December 2019, the virus that causes pneumonia has spread worldwide and became a pandemic. More detailed studies have shown that severe acute respiratory syndrome coronavirus 2 (SARS‐CoV‐2) is the cause of respiratory distress.^1^ Clinico‐epidemiological studies have shown that the most common symptoms of this infection are as follows: fever, cough, fatigue, and shortness of breath.^2^, ^3^ Unfortunately, however, in some people, the disease progresses very quickly and causes acute respiratory distress syndrome (ARDS), which eventually leads to death.^4^, ^5^

Little is known about the pathogenesis of the disease. However, it is clear that when the innate immune system recognizes viral RNAs, three major classes of cytoplasmic recognition receptors are activated: Toll‐like receptors (TLRs), RIG‐I‐like receptors (RLRs), and NOD‐like receptors (NLRs), which trigger expression of interferon (IFN) and activation of antiviral effectors such as natural killer cells, T CD8+ cells, and macrophages;^6^, ^7^, ^8^, ^9^ but uncontrolled host responses lead to aberrant immune cell activation and deregulated cytokine production.^10^ However, the exact molecular mechanism of the pathogenesis of this infection is still unknown. In this study, we investigated the role of MicroRNAs (miRNAs) in the development of respiratory distress. MicroRNAs are small (18–25 nt) RNAs categorized as noncoding RNAs, and their function is to regulate gene expression.^11^ They exhibit nearly 90% sequence homology among humans, mice, and rats.^12^ MiRNAs control gene expression in two ways: by suppressing translation/transcription (RNAi) or by activating transcription (RNAa). Some critical features of miRNAs include tissue specificity, stability, and association with clinicopathological parameters.^13^, ^14^, ^15^ Therefore, differential expression of miRNAs during the progression of COVID‐19 may play an essential role in the host response, and these RNAs may function as biomarkers and even novel therapeutic targets for COVID‐19. To the best of our knowledge, no data have been reported on the expression profile of miRNAs in the bronchoalveolar fluid (BALF) of the lungs of patients with COVID‐19, and all information is based on bioinformatics and in silico studies. The analysis of miRNA profile of patients with COVID‐19 infection would help us to understand the pathogenesis of the disease and find more effective strategies for diagnosis and treatment. Therefore, this study investigated the expression profile of miRNAs in BALF and blood samples from people with COVID‐19 infectious disease.

## 2 METHODS

### 2.1 Sample collection

Blood and bronchoalveolar fluid samples and whole blood were obtained from five severe COVID‐19 patients at Vellayat Hospital Qazvin, IRAN. The patients were informed about the sample collection process and had signed informed consent forms. The recruited patients were between 55 and 65 years old, with severe shortness of breath or breathlessness, rapid and labored breathing, extreme tiredness, and muscle fatigue, who were admitted to the ICU section. These patients had a positive result in the COVID‐19 nasopharyngeal swab RT‐PCR test (*n* = 5). Patients with obesity, diabetes, heart, kidney, and lung diseases were not included in the study due to many factors involved in the expression of miRNAs. Some characterization of the samples is mentioned in Table 1. For BALF preparation, local anesthesia was done with 2% lidocaine injected into the lung segment. 100 ml fractions of room temperature sterile saline were instilled into the right middle lobe or the left lingular segment of the lung. BALF was retrieved by gentle syringe suction and put into sterile containers. Control samples were those with suspected lung cancer who were candidates for lung biopsy. The controls were selected in such a way as to match the gender and age distribution of test patients. Three BALF and blood samples were collected from these control candidates with negative nasopharyngeal swab test results. The Ethics Committee of the Qazvin University of Medical Sciences approved the study.

**TABLE 1:** Characterization of studied samples

ID	Gender	Age	COVID‐19	ARDS	ICU
P1	Male	55	Yes	Yes	Yes
P2	Male	57	Yes	Yes	Yes
P3	Male	51	Yes	Yes	Yes
P4	Female	65	Yes	Yes	Yes
P5	Female	56	Yes	Yes	Yes
C1	Female	58	No	No	No
C2	Male	62	No	No	No
C3	Male	61	No	No	No

### 2.2 Extraction and purification of miRNA

Blood and bronchoalveolar fluid specimens were collected in RNA later RNA Stabilization Reagent (Qiagen GmbH) tubes and then stored at −80°C until further analysis. Total RNA, including miRNAs, was extracted from BALFs and whole blood using TRIZOL reagent (Invitrogen; Thermo Fisher Scientific, Inc.) as described in the manufacturer's protocol. To evaluate the concentration and purity of RNA, we used NanoDrop w ND‐1000. The RNA integrity number (RIN) was assessed using the Agilent 2100 Bioanalyzer with RNA 6000 series II Nano Lab Chip analysis kit (Agilent Technologies, Inc.). The 2100 Bioanalyzer generates numerical RIN values. RIN is an incremental scale spanning 0–10, with 10 indicating maximum RNA integrity. All RNA samples extracted from BALFs and blood samples were of high integrity as judged by the obtained RIN values of higher than eight. Then, all samples with a ratio (28S/18S) of above 1.8, OD 260/280 ratio greater than 1.9, and RIN >8 were sent to BGI company in China for sequencing. In this regard, small RNA libraries were generated from the purified RNA using the Illumina Small RNA v1.5 sample preparation kit (Illumina, Inc.), according to the manufacturer's instructions.

### 2.3 miRNA library construction and sequencing

In the first step, the Illumina Small RNA v1.5 sample preparation kit (Illumina, Inc.) was used for the purification of small RNA, according to the manufacturer's instructions. Each RNA sample was size‐fractionated, and 18–30 nt RNA was isolated and purified. In the next step, 5′ and 3′ adaptor ligation was done. RNA was reverse‐transcribed and amplified using 14 PCR cycles of 98°C for 10 s and 72°C for 15 s to generate small RNA libraries, and then the library was performed to the numbers of the studied samples. Average molecule length and purity were evaluated by Agilent Technologies 2100 Bioanalyzer, and quantitative PCR was performed for detection of the concentration, using EvaGreen® dye (Jena Bioscience). The validated libraries were subsequently sequenced on an Illumina HiSeq 2000 (Illumina, Inc.).

### 2.4 Analysis of sequenced data

In the first step, Cutadapt software (version 2.10; https://cutadapt.readthedocs.io/en/stable/) was used for adaptor trimming, low‐quality reads, rRNA, and tRNA removal. The rRNA proportion less than 5% showed that the studied samples had good quality. Bowtie software (version 1.2.3; www.sourceforge.net/projects/bowtie‐bio/files/bowtie) was used to map miRNA data to the miRBase (version 22; http://www.miRbase.org/). FastQC (version 0.11.9) and multiQC (version v1.8) were used for quality control of the raw read data. Our raw data quality was good, and the quality score for each sample was between 33 and 37. For miRNA‐seq data, two peaks at 22 nt and 33 nt were observed in the read length distribution.

### 2.5 Quantitative reverse transcription‐polymerase chain reaction (qRT‐PCR)

To confirm miRNA sequencing results, the RNA samples of patients with ARDS (*n* = 25) and controls (*n* = 15) were used; moreover, the expression of miRNAs and their target genes, which had the highest score and significance, examined by the Real‐Time PCR method. We used TRIZOL reagent to isolate the total RNA from the samples based on kit protocols (Invitrogen Life Technology Co). Three steps of phenol/chloroform purification were done to separate the proteins. To evaluate the concentration and purity of RNA, we used NanoDrop w ND‐1000.

The miRCURY LNA Universal RT microRNA kit (Exiqon) and Revert Aid First Strand cDNA Synthesis Kit (Thermo Scientific, Fermentas) were used to reverse the transcription of RNA. The final volume was 10 μl containing 1 ng/μl of purified total RNA, 5× reaction buffer, Enzyme mix, and nuclease‐free water. The mixture was incubated at 42°C for 60 min and then at 95°C for 5 min (for enzyme inactivation). It was then quickly cooled to 4°C. The primers of 8 miRNAs (miR‐4284, miR‐155‐5p, miR‐15a‐5p, miR‐548c‐5p, miR‐15b‐5p, miR‐18a‐3p, miR‐21‐5p, and miR‐14a‐3p) and their internal control (miR‐miR30a‐5p) were ordered and synthesized by Qiagen Company (Qiagen) in the form of LNA. Target genes (HDAC, smad‐7, CRP, IL‐12, ERK‐1, HOX‐1, CCL‐20, and TGF‐β) primers are mentioned in Table 2. Also, we used beta‐actin as an internal control. Subsequently, real‐time quantification was performed using Rotor gene‐Q real‐time PCR system (Qiagen). Each real‐time PCR (10 μl) included 1 μl of reverse and forward primers (Qiagen), 5 μl of Ampliqon real Q plus 2× master mix green (Ampliqon), and 4 μl of diluted cDNA. The reactions were incubated in a 72‐well optical strip at 95°C for 15 min (enzyme activation), followed by 95°C for 20 s and 60°C for 60 s (40 cycles). All the reactions were run in triplicate. After the reactions, the mean Ct was determined from the triplicate PCRs. We used Ct values to evaluate the expression levels of the miRNAs and target genes. The expression value of miRNAs relative to internal controls was determined using the 2^‐△Ct^ method.

**TABLE 2:** Primer sequence of studied target genes

Target	Forward primer sequence	Reverse primer sequence
TGF‐β	5′‐TACCTGAACCCGTGTTGCTCTC‐3′	5′‐GTTGCTGAGGTATCGCCAGGAA‐3′
ERK‐1	5′‐CCTGCGACCTTAAGATTTG TGATT‐3′	5′‐CAGGGAAGATGGGCCGGTTA GAGA‐3′
CRP	5′‐GAACTTTCAGCCGAATACATCTTTT‐3′	5′‐CCTTCCTCGACATGTCTGTCT‐3′
HDAC	5′‐CAACGAATGAATGAACAGCC‐3′	5′‐CATCTCCTCAGCATTGGCTT‐3′’
Smad‐7	5′‐TGTCCAGATGCTGTGCCTTCCT‐3′	5′‐CTCGTCTTCTCCTCCCAGTATG‐3
CCL‐20	5′‐GTGGGTTTCACAAGACAGATGGC‐3	5′‐CCAGTTCTGCTTTGGATCAGCG‐3
IL‐12	5′‐ACGAGAGTTGCCTGGCTACTAG‐3	5′‐CCTCATAGATGCTACCAAGGCAC‐3
HOX‐1	5′‐CAGCGCAGACTTTTGACTGGATG‐3	5′‐TCCTTCTCCAGTTCCGTGAGCT‐3

### 2.6 Bioinformatics analysis

For bioinformatics analysis of the sequenced miRNAs, we used miRWalk (http://miRwalk.uni‐hd.de) and miRBase (http://www.miRbase.org) database. Gene Ontology (GO) was performed using multiple tools and algorithms for identifying miRNA‐target genes and their corresponding pathways. GO pathway enrichment analysis of biological processes was applied for the predicted miRNA‐target genes, and the human genome was a reference set. We used the z test to determine GO terms or Kyoto Encyclopedia of Genes and Genomes (KEGG) pathway enrichment. Furthermore, the Database for Annotation, Visualization and Integrated Discovery (DAVID) bioinformatics tools (http://david.abcc.ncifcrf.gov) were employed to thoroughly examine the functional characterization results of the enriched cognate mRNA target genes via comparative analysis of biological functions, enrichment analysis of signaling pathways, and assignment of the disease categories. Significance P‐values for DEGs were calculated by Student's *t* test. For identifying miRNA–mRNA interaction, we used multiMiR R package (version 2.3; http://multimiR.org), and then miRNA‐mRNA networks were constructed with Cytoscape (version 3.7.2; https://cytoscape.org/). Each miRNA‐mRNA pair was filtered using the most stringent criteria.

## 3 RESULTS

RNA sequencing method was used to evaluate the changes in microRNA expression in BALF and blood samples of patients with COVID‐19 infection (ARDS) and controls. Nearly, 20,559,321 clean high‐quality reads were obtained among the 21,221,234 effective reads (97%). The frequencies of the obtained products length were as follows: 21 nt (6.2%), 22 nt (62.1%), 23 nt (31.7%), and 24 nt (1.1%). The rate of degradation was very insignificant because the contribution of rRNA in the reads was <0.29%.

### 3.1 Differential expression of miRNAs in BALFs and blood

**FIGURE 1:**
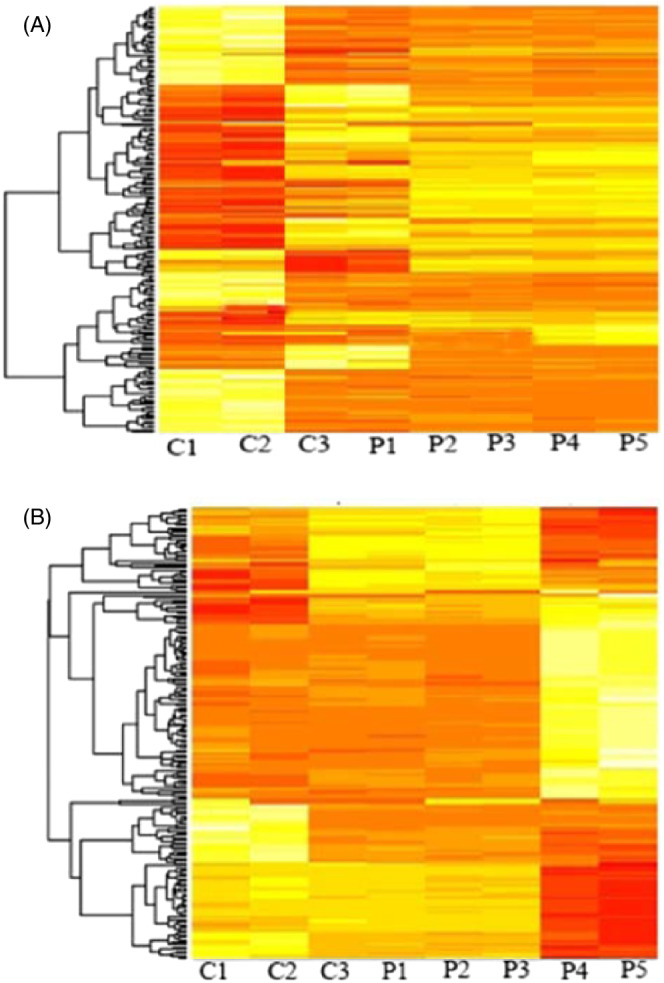
Heat map of genes significantly upregulated and downregulated in COVID‐19 patients (A) BALF and (B) whole blood compared with controls (Fold change >2)

Our results showed that differentially expressed miRNAs were higher in BALF samples than in blood samples. To select the differentially expressed miRNAs more accurately, we used fold change cut‐off values of <2.0 and >2.0. Then, three significant levels were chosen for analysis *p* < 0.05, *p* < 0.01, and *p* < 0.001. In blood, a total of 2234 differentially expressed miRNAs were found, in which 543 miRNAs are significantly upregulated while 220 miRNAs are downregulated. In BALF samples, 8223 differentially expressed miRNAs were identified, in which 825 miRNAs are significantly upregulated while 432 miRNAs are downregulated. The differentially expressed miRNAs are represented in a scaled heat map; it compares patients and healthy controls for both BALF and blood samples (Figure 1A,B).

The 10 most differentially expressed miRNAs in infected blood and BALF samples (up and downregulated) are compared with uninfected samples, as shown in Tables 3, 4, 5, 6. As mentioned in Table 3, miR‐4284 was the most upregulated miRNA in infected BALF samples compared with uninfected BALFs (2^‐∆ct^: 11.1235, *p*: 0.0000321).

**TABLE 3:** Top upregulated miRNAs in BALF samples of ARDs patients in comparison with normal samples

miRNA	No. of reads	2^‐∆ct^	*p*‐Value
mir‐4284	99.011/213.321	11.1235	0.0000321
mir‐155‐5p	6.0021/4.21	8.7653	0.000153
mir‐4485‐3p	3.321/1.991	4.123009	0.0001134
mir‐483‐3p	204.10/1.32	3.00043	0.0012
mir‐6891‐5p	121.08/154	2.8754	0.010322
mir‐200c	110.11/124	2.0043	0.02354
mir‐4463	32.01/102	1.99544	0.0123
mir‐155‐3p	3/102	1.87003	0.00123
mir‐483‐5p	11/001	1.8735	0.01256
mir‐98‐5p	9/012	1.8023	0.0001234

**TABLE 4:** Top downregulated miRNAs in BALF samples of ARDs patients in comparison with normal samples

miRNA	No. of reads	2^‐∆ct^	*p*‐Value
miR‐15a‐5p	96.765/124.654	0.000011	0.0004
miR‐548c‐5p	65.764/143.143	0.000123	0.025523
miR‐548d‐3p	19.543/1.543	0.000123	0.000164
miR‐365a‐3p	121.98/12.554	0.007543	0.00343
miR‐3939	119.10/654	0.0347	0.00021
miR‐514b‐5p	32.131/543	0.01256	0.01231
miR‐513a‐3p	10.154/343	0.017	0.009832
miR‐513a‐5p	15/234	0.12764	0.000432
miR‐664a‐3p	10/32	0.275438	0.0765
miR‐766‐3p	4/321	0.37598	0.0143

**TABLE 5:** Top upregulated miRNAs in blood samples of ARDs patients in comparison with normal samples

miRNA	No. of reads	2‐Δct	*p*‐Value
miR‐15b‐5p	93.122/213.321	5.2456	5.95E‐05
miR‐18a‐3p	3.223.7/7.112	3.960113	0.00453
miR‐486‐3p	2.485.2/3.012	3.20032	0.000168
miR‐486‐5p	324.12/1.554	2.088128	0.0022
miR‐146a‐5p	143.18/654	1.95761	0.01432
miR‐16‐2‐3p	113.12/567	1.872749	0.038765
miR‐6501‐5p	21.09/112	1.832143	0.005301
miR‐3605‐3p	4/158	1.71453	0.0001381
miR‐618	15/113	1.70001	0.00232
miR‐623	11/027	1.69871	0.00098

**TABLE 6:** Top downregulated miRNAs in blood samples of ARDs patients in comparison with normal samples

miRNA	No. of reads	2^‐∆ct^	*p*‐Value
miR‐21‐5p	15.123/3.143	0.003241	2.11E‐01
miR‐142a‐3p	9.543/123.1	0.001432	0.00564
miR‐181‐a	178.12/1.554	0.002764	0.0123
miR‐31‐5p	113.10/123	0.02419	0.00032
miR‐99a‐5p	102.11/654	0.012334	0.02543
miR‐342‐5p	12.00/102	0.011	0.000101
miR‐183‐5p	2/142	0.23412	0.000032
miR‐627‐5p	11/100	0.2784323	0.0564
miR‐144‐3p	7/021	0.543216	0.0987

MiR‐15a‐5p was the most downregulated miRNA in infected BALFs in comparison with noninfected BALF (2^‐∆ct^:0.000011, *p*: 0.0004) (Table 4). Besides, in infected blood samples, miR‐15b‐5p was the most upregulated miRNA compared with control blood samples (2^‐∆ct^: 5.2456, *p*: 5.95E‐05) (Table 5). Furthermore, miR‐21‐5p was the most downregulated miRNA in infected blood (2^‐∆ct^: 0.003241, *p*: 2.11E‐01) (Table 6).

### 3.2 Functional enrichment analysis of the targeted genes of miRNAs

Since the molecular mechanisms involved in the pathogenesis of the COVID‐19 virus are not yet known, we used the web‐based GO analysis bioinformatics resources tool of DAVID v6.8 (https://david.ncifcrf.gov/).

**FIGURE 2:**
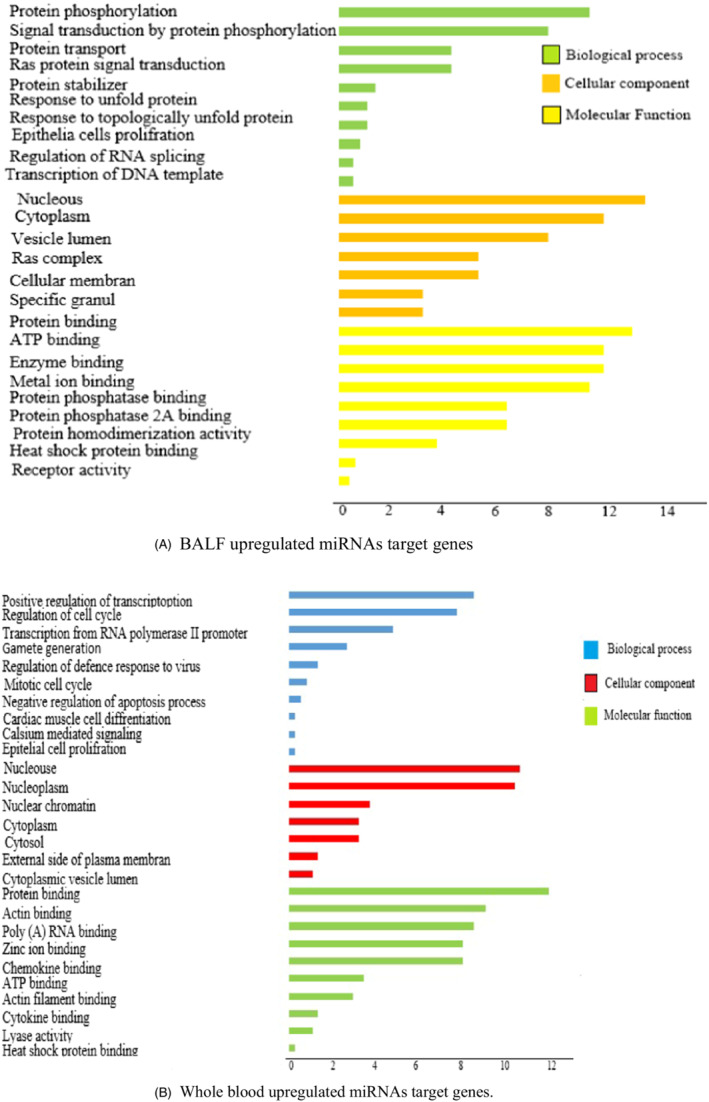
GO term pathway enrichment (Biological process, Cellular component, Molecular function) of upregulated and downregulated miRNAs target genes expression in BALF and whole blood of COVID‐19 patients. (A) BALF upregulated miRNAs target genes (B) Whole blood upregulated miRNAs target genes

Based on the top differentially expressed miRNAs in BALF and blood samples, the biological and molecular processes involved in COVID‐19 infection pathogenesis were globally assessed. We used MiR Tar Base 2020 database (http://miRTarBase.cuhk.edu.cn/) to find the target genes of miRNAs. Hypergeometric Distribution Mathematical Model was used to detect the *p*‐value of GO. The targets were enriched in the GO when the *p*‐value was < 0.05. In this regard, the top major miRNAs were enriched in the GO (biological process, BP; cellular component, CC; molecular function, MF). Virus infection causes changes in gene expression in specific cellular biological processes. In this regard, we performed miRNAs targeted genes functional enrichment analysis in BALF and blood samples to monitor the cellular changes of patients and control samples. In infected BALF samples, the targeted upregulated genes resulting from the downregulation of miRNAs are associated with the regulation of inflammatory response and apoptosis process (Figure 2A). However, upregulated targeted genes in the blood are mainly enriched in the processes associated with the activation of humeral, cellular immunity, and response to the virus (Figure 2B).

**FIGURE 3:**
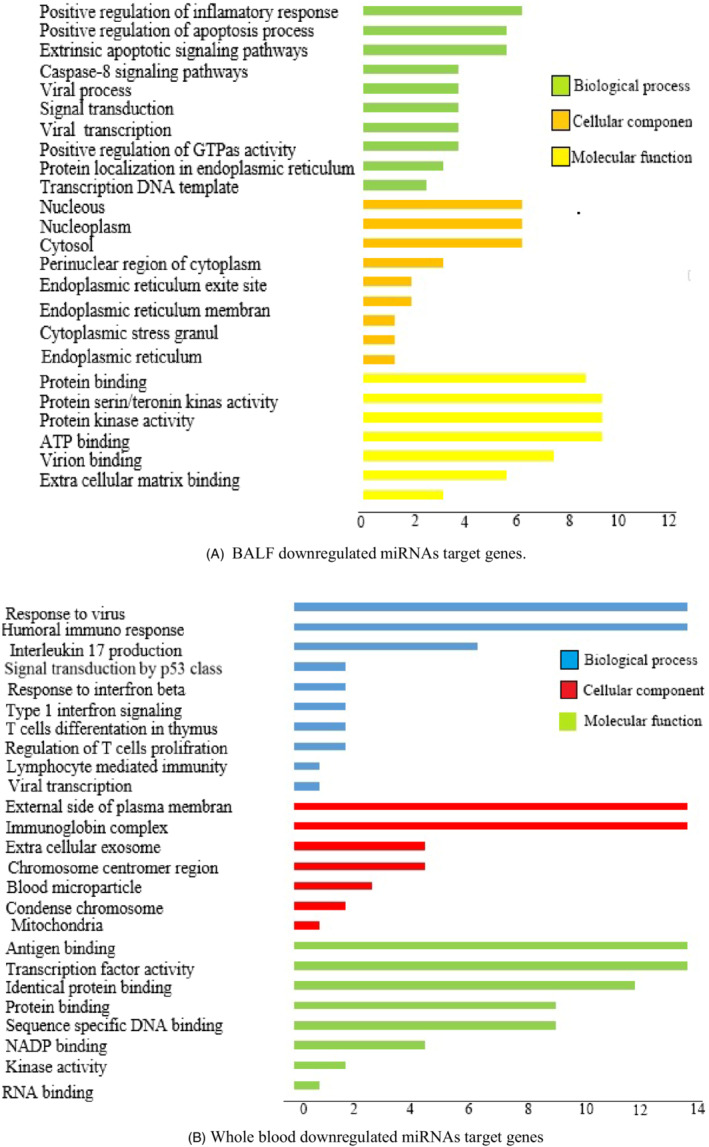
GO term pathway enrichment (Biological process, Cellular component, Molecular function) of upregulated and downregulated miRNAs target genes expression in BALF and whole blood of COVID‐19 patients. (A) BALF downregulated miRNAs target genes (B) Whole blood downregulated miRNAs target genes

Interestingly, the downregulated targeted genes in infected BALF samples resulting from upregulation of miRNAs are enriched in biological processes, which involve positive regulation of cell signaling pathways. These processes include protein phosphorylation, transport, and stabilization, indicating that cellular signaling pathways in the BALF are out of control (Figure 3A). In contrast, the decreased genes in patients' blood samples are associated with other biological processes such as cell cycle regulation and gamete generation. (Figure 3B).

### 3.3 Comparison of detailed KEGG data analysis of infected BALF and blood

**FIGURE 4:**
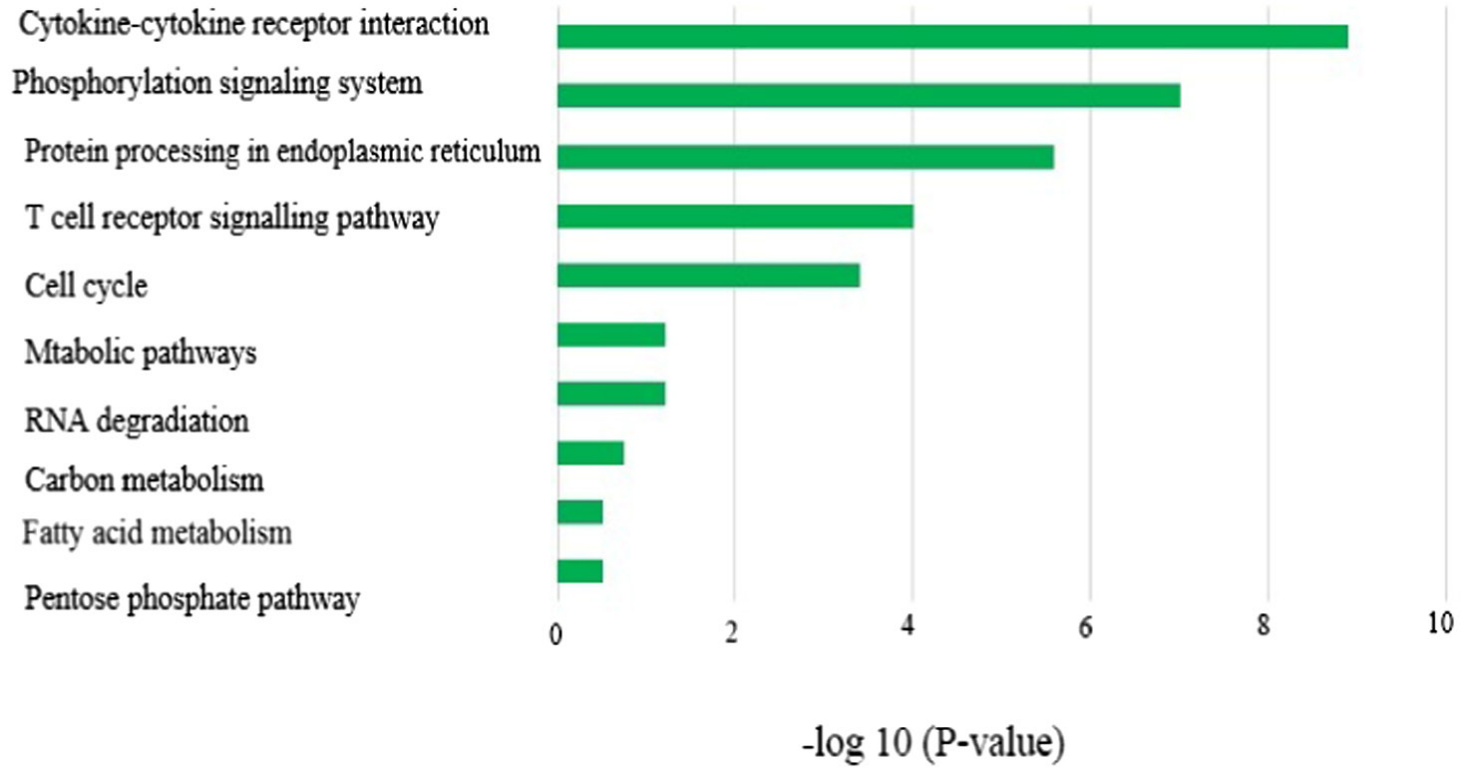
Kyoto Encyclopedia of Genes and Genomes pathway of upregulated miRNAs target genes in BALF and blood samples

We used KEGG release 76.0 application for KEGG (https://www.genome.jp/kegg/) pathway analysis. In this regard, the same enrichment rule was applied as in GO, when the P‐value and the Q‐value were < 0.05. The KEGG pathway studied was considered enriched and included in the results for both infected BALF and blood comparisons. Cytokine–cytokine receptor interaction and T‐cell receptor signaling pathway were found to be enriched, among others, in infected blood and BALF samples compared with control (Figure 4).

### 3.4 Results of the miRNA‐mRNA correlation study

**FIGURE 5:**
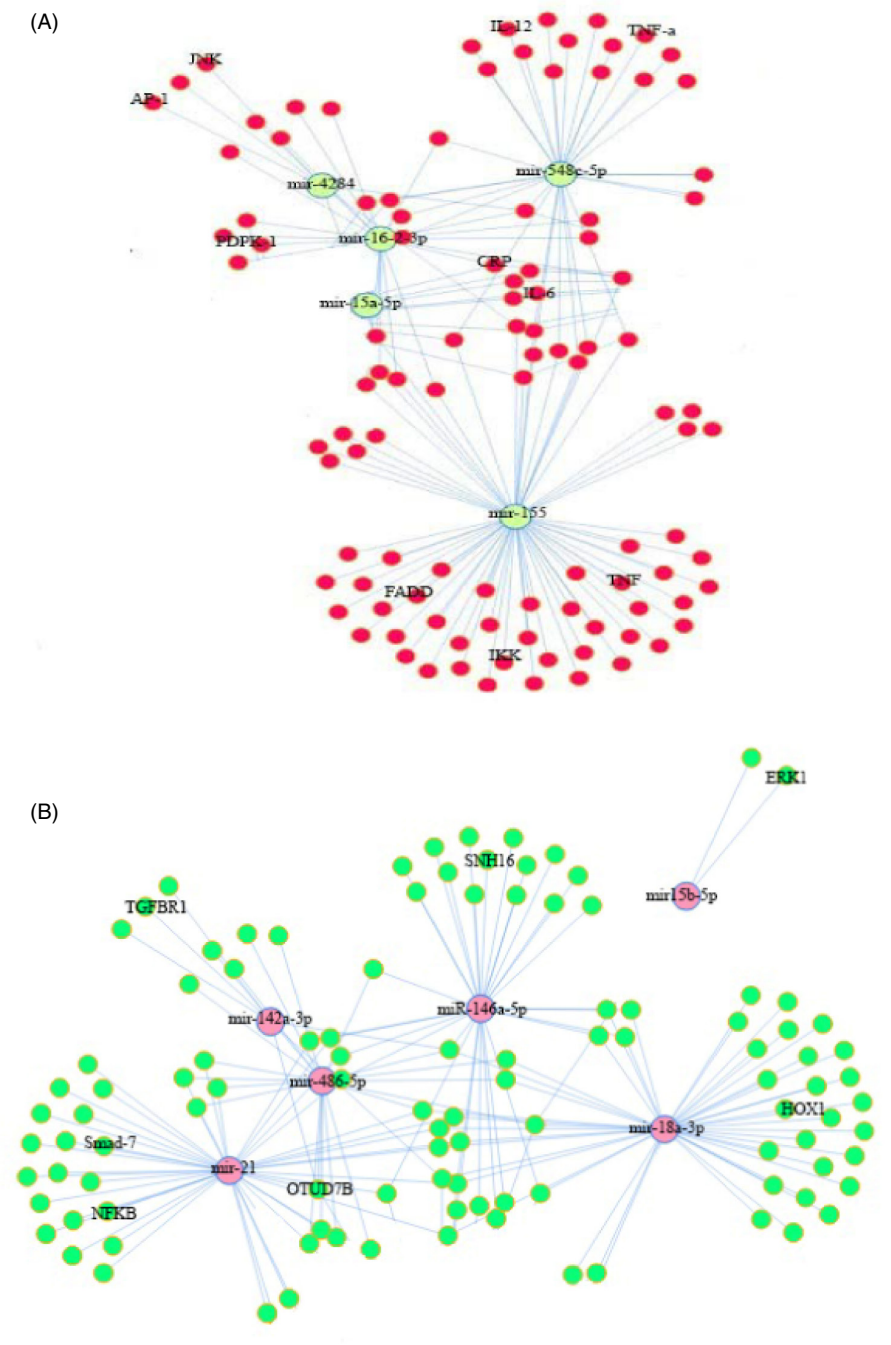
(A) Top differentially expressed miRNA‐mRNA network in BALF samples. (B) Top differentially expressed miRNA‐mRNA network in whole blood samples. Only pairs mentioned in the text were labeled

To better understand the function of the studied miRNAs, we analyzed the miRNA‐mRNA correlation. In this regard, the validated miRNA‐target genes list obtained from miRTarBase contained 5872 miRNA‐target genes interaction. These miRNA‐target genes interaction pairs were obtained by combining 40 high‐score miRNAs in BALFs, blood, and 232 target genes in miRTarBase. Among these, we found 1560 miRNA‐ target genes interaction pairs in which both miRNA and mRNA were significantly expressed (*p* < 0.001) and then constructed an integrated miRNA‐mRNA regulatory network with MIENTURNET package (Figure 5A,B). Based on our study, differentially expressed miRNAs in infected BALFs and blood samples promote the expression of the mRNAs that activate the immune, inflammatory, and apoptosis pathways. This section of the results explained the target mRNAs of two miRNAs that had the highest score and significance in BALF and blood samples.

In BALF samples, upregulation of miR‐4284 promotes apoptosis with underexpression of MLL, BCL‐10, and HDAC.^16^ Upregulated miR‐155‐5p targeted smad‐7 and promoted the expression of inflammation‐related genes.^17^ Also, upregulated miR‐155‐5p participates in the activation of TNF‐α and IL‐6, by eliciting the Fas‐associated death domain protein (FADD), Ik B kinase ε (IKKε), and receptor (TNF receptor superfamily) interacting serine–threonine kinase 1 or RIPK1 (18). Regarding the downregulated miRNAs in BALF samples, our investigation showed that underexpression of miR‐15a‐5p could raise the levels of CRP, IL‐6, and ICAM1. CRP is a sensitive biomarker for systemic inflammation.^18^, ^19^ Another downregulated miRNA was miR‐548c‐5p, which acts as an anti‐inflammatory factor. Downregulation of this miRNA causes increased levels of inflammatory cytokines such as IL‐12, TNF‐α, and less nuclear translocation of pNF‐κB in pTHP1 cells.^20^

In blood samples, we showed upregulation of miR‐15b‐5p with a high score. These miRNAs may target extracellular signal‐regulated kinase 1 (ERK‐1), inhibit cell proliferation, and induce apoptosis.^21^ The second upregulated miRNA was miR‐18a‐3p, which may target the 3’UTR region of the HOXA1 gene, thus blocking its expression and triggering apoptosis.^22^ The first downregulated miRNA was miR‐21‐5p; it is associated with elevated inflammatory immune responses by targeting smad‐7.^23^ The miR‐142a‐3p was the second downregulated miRNA that can target TGFBR1 and diminish TGF‐β signaling. This may indicate a novel pathogenic pathway that simultaneously decreases the immunomodulatory effects of cytokine, and downregulation of this miRNA leads to an increased immune and cytokine response.^24^

### 3.5 Confirmation of RNA‐seq results by QRT‐PCR method

**FIGURE 6:**
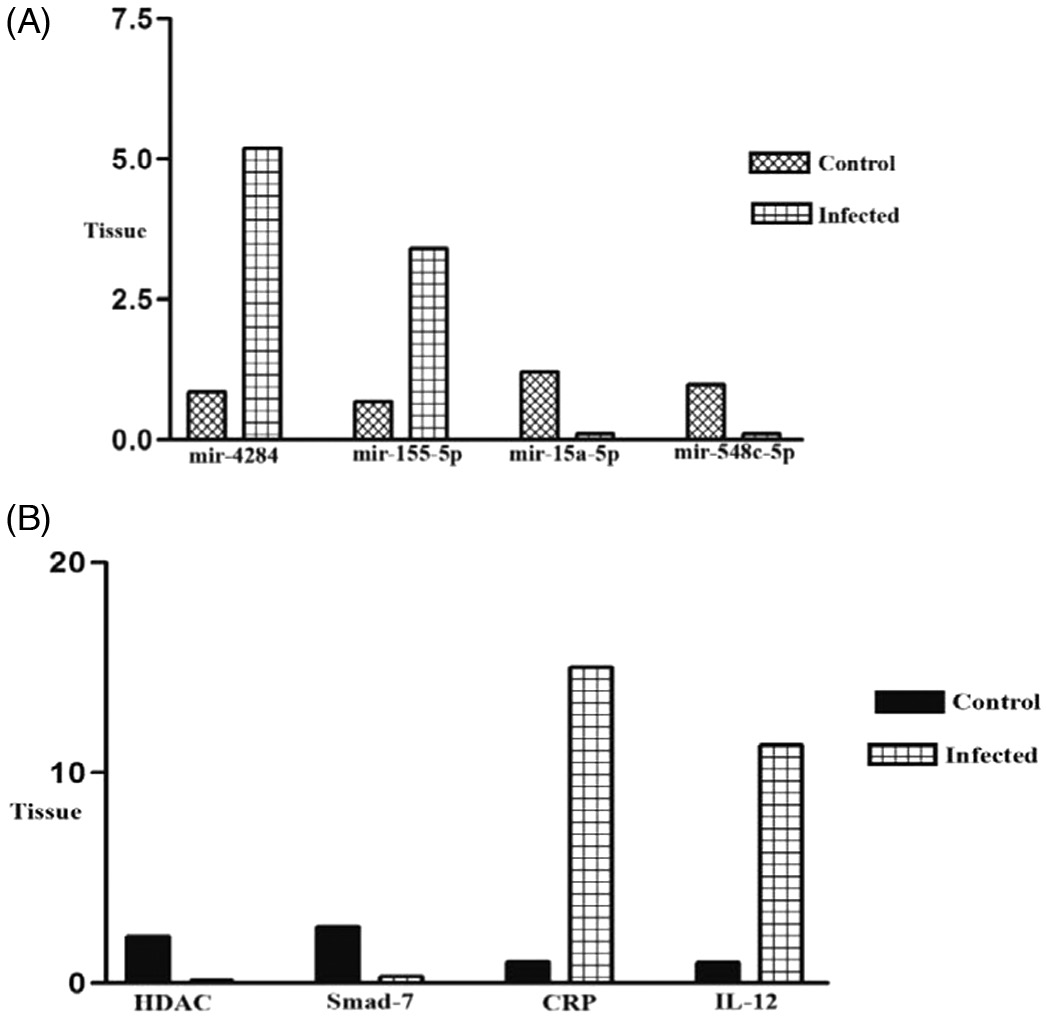
(A) Expression rate of miR‐4284, miR‐155‐5p, miR‐21‐5p, miR‐15a‐5p, and miR‐548c‐5p (four top differentially expressed miRNAs in BALF samples) in infected patients in comparison with controls (*p* < 0.001). (B) The expression rate of HDAC, Smad‐7, CRP, and IL‐12 (four top differentially expressed targeted genes of above miRNA in BALF samples) in infected patients in comparison with controls (*p* < 0.0001)

**FIGURE 7:**
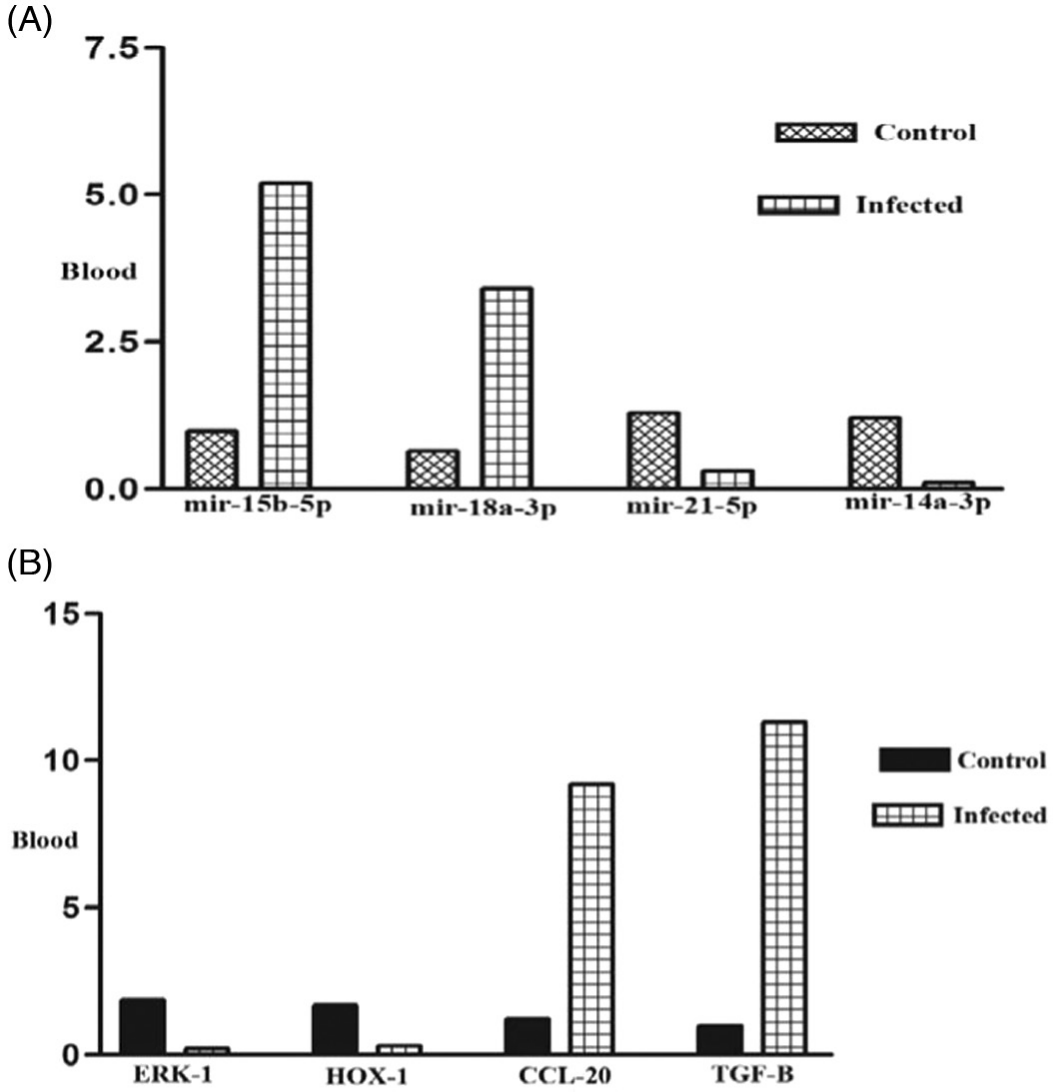
(A) Expression rate of miR‐15b‐5p, miR‐18a‐3p, miR‐21‐5p, and miR‐14a‐3p (four top differentially expressed miRNAs in blood samples) in infected patients in comparison with controls (*p* < 0. 01). (B) The expression rate of ERK‐1, HOX‐1, CCL‐20, and TGF‐B (four top differentially expressed targeted genes of above miRNAs in BALF blood samples) in infected patients in comparison with controls (*p* < 0.0001)

To confirm RNA‐seq results, we selected 8 high‐score (up and downregulated) miRRNA and their target genes in BALF and blood samples. About the BALF samples, our results showed significant upregulation of miR‐4284 (*p* < 0.001) and miR‐155‐5p (*p* < 0.01) (Figure 6A) and downregulation of their target genes HDAC (*p* < 0.001) and smad‐7 (*p* < 0.0001) (Figure 6B). Also, miR‐15a‐5p (*p* < 0.01) and miR‐548c‐5p (*p* < 0.001) BALF (Figure 6A) and their target genes CRP (*p* < 0.00001) and IL‐12 (*p* < 0.01) showed significant downregulation and upregulation in BALF samples, respectively. (Figure 6B). Our results showed significant upregulation of miR‐15b‐5p (*p* < 0001) and miR‐18a‐3p (*p* < 0001) in blood samples (Figure 7A). The target genes of these miRNA (ERK‐1 (*p* < 0001) and Hox‐1 (*p* < 0.01)) showed a significant downregulation (Figure 7B).

We also showed significant downregulation of miR‐21‐5p (*p*< 0.001) and miR‐14a‐3p (*p* < 0.0001) (Figure 7A) and upregulation of CCL‐20 (*p* < 0.001) and TGF‐β (*p* < 0.0001) in infected blood samples (Figure 7B). The results of the correlation study between miRNA and the expression of its target genes expression showed a significant negative correlation, which is listed in Table 7.

**TABLE 7:** Shows correlation between RNA expression rate of miRNA and their target genes

RNA expression rate	*R*	Sig.
MiR‐4284, HDAC	*r* = −0.48	<0.0001
MiR‐155, Smad‐7	*r* = −0.63	<0.0001
MiR‐15a‐5p, CRP	*r* = −0.24	<0. 0012
MiR‐548C, IL‐12	*r* = −0.38	<0. 0025
MiR‐15b, ERK‐1	*r* = −0. 58	<0.001
MiR‐18a, HOX‐1	*r* = − 0.68	<0.001
MiR‐21‐5p, CCL‐20	*r* = −0.69	<0.0001
MiR‐14‐a, TGF‐B	*r* = −0.39	<0.0001

## 4 DISCUSSION

The COVID‐19 pandemy started in 2019 and is still going on. In general, infection with SARS‐CoV‐2 causes this disease and usually begins with cold symptoms such as fever, sore throat, and headache. If left untreated, it enters the inflammatory phase, and lung involvement occurs.^25^ The definitive treatment of this disease has not yet been known because the pathogenesis is not fully understood. Designing novel therapeutics approaches is difficult because our understanding of the host immune response to SARS‐CoV‐2 infection is limited. Studies have shown that viral infections alter transcriptome expression in host cells and ultimately provide the right conditions for the virus replication.^26^, ^27^, ^28^ In this study, the expression profiles of the miRNAs in the lung BALF and blood samples of the patients with ARDS were examined by the RNA‐seq method. We showed that 2234 and 8223 miRNAs were differentially expressed in blood and BALF samples of ARDS patients. Among these miRNAs, miR‐21‐5p and miR‐15b‐5p were differentially expressed in blood samples with high significant scores.

In BALF samples, miR‐4284 and miR‐15a‐5p had high scores. Generally, inflammatory responses happen to fight the virus. Still, when the expression of genes involved in this pathway is abnormally high, it leads to a “cytokine storm,” which finally manifests itself in the form of pneumonia and pulmonary fibrosis in ARDS patients.^29^, ^30^, ^31^ Based on our results, miR‐21‐5p was one of the downregulated miRNAs in ARDS patients' blood samples with the highest score. There are different hypotheses about the role of this miRNA in the immune response and inflammation process. One of the target genes of this miRNA is the Smad‐7 gene, which plays an essential role in the inflammatory pathway.^23^

Downregulation of miR‐21‐5p may increase Smad‐7 gene expression, which plays a vital role in fibrotic conditions, and its signaling pathway is regulated by Smad molecules, including Smad‐7.^23^, ^24^ In this regard, Sheedy (2015) has shown that inflammatory immune responses are elevated with downregulation of miR‐21‐5p in leucocytes, as it could lead to the activation of the NF‐κB signaling pathway. Furthermore, miR‐21‐5p suppresses the TGF‐β expression, promoting T17 cell differentiation and T‐cell‐mediated inflammation.^32^ Another hypothesis is that miR‐21‐5p may also directly target *CCL‐20* and *MYC*, whose overexpression fosters the inflammatory response and the T‐cell metabolic reprogramming.^33^ Also, Tang et al.^34^ 2020, introduced miR‐21‐5p as a novel biomarker of the COVID‐19 infection. MiR‐15b‐5p was one of the highest score upregulated miRNAs in blood samples. This miRNA can directly bind to the SARS‐CoV‐2 genome, and repressing the expression of *IFNG* and *CD69* can induce T‐cell expansion.^35^ This miRNA might also target extracellular signal‐regulated kinase 1 (ERK‐1) by binding to its 3′‐untranslated region (3’‐UTR), inhibiting cell proliferation, and inducing apoptosis.^35^, ^36^, ^37^ These pieces of evidence suggest a crucial role for miR‐15b‐5p in COVID‐19 pathogenesis.

In BALF samples, miR‐15a‐5p with the highest score was selected; this miRNA is one of the five miRNAs that commonly bind to SARS‐CoV, MERS‐CoV, and SARS‐CoV‐2.^19^ It was one of the most essential miRNAs in this study because it targets two critical genes in the path of inflammation and apoptosis (CRP, IL‐6). C‐reactive protein (CRP) is a protein made by the liver sent into the bloodstream in response to inflammation. It has been shown that CRP levels are correlated with levels of inflammation. CRP levels can promote phagocytosis and activate the complement system.^38^, ^39^ Transcriptional induction of the CRP gene primarily occurs in hepatocytes in response to increased inflammatory cytokines, especially interleukin‐6 (IL‐6) with IL‐1 enhancing the effect.^39^ In this line, analysis of CRP and other cytokines shows predictive value for the severity of COVID‐19. High levels of CRP and IL‐6 (a hepatic inducer of CRP) and IL‐10 have been used as predictive factors for COVID‐19.^39^ High levels of CRP have been associated with mortality from this infection, and CRP has been identified as a molecule capable of causing damage during SARS‐CoV‐2 infection.^40^, ^41^ CRP induces apoptosis by several mechanisms: (1) induction of pro‐apoptotic cytokines such as TNF‐α and IL‐1‐β and induction of reactive oxygen species, (2) induction of p53 upregulation, (3) activation of genes related to the expression of adhesion molecules and chemotactic cytokines, (4) induction of GADD153 gene expression related to cell cycle arrest and DNA damage.^38^ Another miRNA was miR‐4284, which was significantly downregulated in the BALF samples. Typically, this miRNA can elevate MLL, BCL‐10, and HDAC, which play a role in blocking apoptosis and increasing proliferation. In our study, we hypothesized that upregulation of miR‐4284 promotes apoptosis with underexpression of MLL, BCL‐10, and HDAC.^16^

**FIGURE 8:**
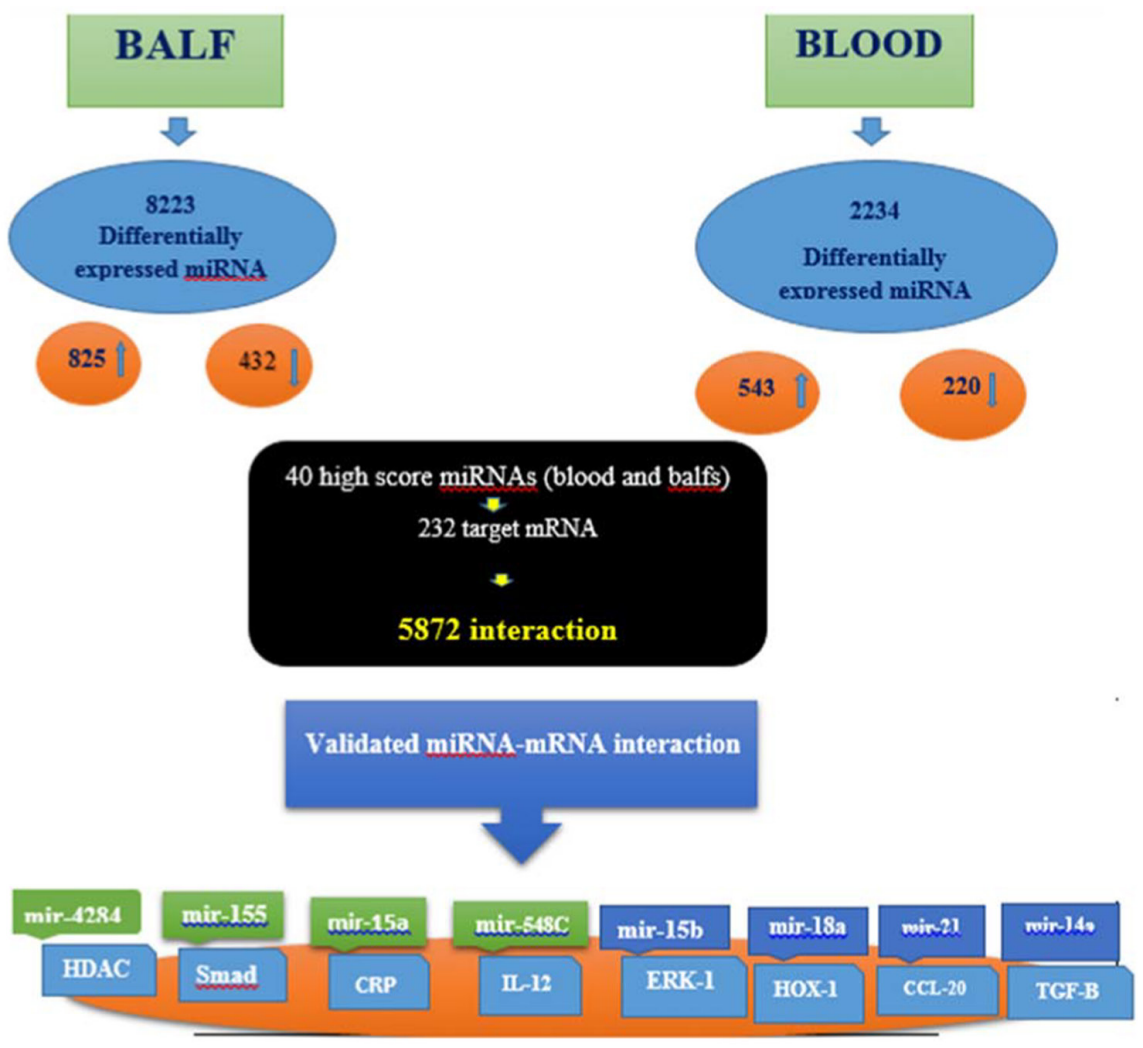
Workflow of the study. In this research, BALF and blood samples of patients with ARDS were investigated. Data from miRNA experiments obtained from three normal and five infected samples. Total numbers of differentially expressed miRNAs in BALF was 8223 and in blood was 2223, which in BALF 825 were upregulated and 432 were downregulated. In blood samples, 543 miRNAs were upregulated and 223 miRNA downregulated. miRTarBase results for 40 high‐score differentially expressed miRNAs showed 232 target mRNA, which have 5872 interactions. The knowledge base was further validated with wet‐lab experiment, where we analyzed the correlation between some miRNAs and their target genes

Based on the results of this study and the potential role of miRNAs in the pathogenesis of COVID‐19 disease, it can be speculated that it is possible to use miRNAs as a target for the treatment. Since the expression of miRNAs is affected by various factors, we tried to eliminate interfering factors as much as possible. Patients were selected at approximately the same age range, and patients with a history of diabetes, heart, kidney, and lung disease were excluded from the study. Still, the rest of the interventions were out of control. The current study showed that differentially expressed microRNA (miRNA) in blood and bronchoalveolar fluid (BALF) samples of acute respiratory distress syndrome (ARDS) patients is involved in activating the inflammatory and apoptosis process; workflow of this research is shown in Figure 8.

## FUNDING INFORMATION

This research was supported by the Cellular and molecular research center of the Qazvin University of Medical Science.

## CONFLICT OF INTEREST

The authors declare that there is no conflict of interest.

## Data Availability

The data supporting the results of this study are available from the corresponding author upon request [SM]. The data are not publicly available because they contain information that could compromise the privacy of the research participants.
